# Stunted African toddlers digest and obtain energy from energy-dense thick sorghum porridge

**DOI:** 10.1038/s41430-025-01632-y

**Published:** 2025-05-23

**Authors:** Fatimata Cisse, Clay Swackhamer, Hawa G. Diall, Atossa Rahmanifar, Mariam Sylla, Antone R. Opekun, Michael A. Grusak, Amy H-M. Lin, Elizabeth A. Pletsch, Anna M. R. Hayes, Roberto Quezada-Calvillo, Buford L. Nichols, Bruce R. Hamaker

**Affiliations:** 1 Whistler Center for Carbohydrate Research, Department of Food Science, Purdue University, West Lafayette, IN 47906 USA; 2 Institut d’Economie Rurale du Mali (IER), BP 258 Bamako, Mali; 3 Département de Pédiatrie, Centre Hospitalier et Universitaire Gabriel Toure, BP 267 Bamako, Mali; 4 Nutrition Consultant, West Lafayette, IN 47906 USA; 5 Departments of Medicine & Pediatric Sections of Gastroenterology and G.I., Baylor College of Medicine, Houston, TX 77030 USA; 6 USDA-ARS Children’s Nutrition Research Center, Houston, TX 77030-2600 USA; 7 Facultad de Ciencias Químicas, Universidad Autónoma de San Luis Potosí, Zona Universitaria, San Luis Potosí, 78210 Mexico; 8 Present Address: Nutrition Program, School of Nutrition and Public Health; Department of Food Science and Technology; Oregon State University, Corvallis, OR 97333 USA; 9 Present Address: USDA-ARS Edward T. Schafer Agricultural Research Center, Fargo, ND 58102 USA; 10 Present Address: Singapore Institute of Food and Biotechnology Innovation, Agency for Science, Technology and Research, Singapore, Singapore; 11 Present Address: USDA-ARS Beltsville Human Nutrition Research Center, Beltsville, MD USA; 12 Present Address: Department of Food Science and Technology, Oregon State University, Corvallis, OR 97333 USA

**Keywords:** Translational research, Feeding behaviour

## Abstract

**Background/objectives:**

Increasing the energy density of porridges could help meet the needs of moderately malnourished, stunted children. However, it is not clear whether stunted toddlers can adequately digest and obtain energy from energy-dense porridges with thick texture. A clinical study was conducted in Bamako, Mali, using ^13^C-labeled substrates and serial breath sampling to determine whether stunted toddlers differed from healthy toddlers in their capacity to digest thick and thin sorghum porridges.

**Subjects/methods:**

Experimental porridges, including a traditional porridge (control), a starch-enriched calorie-dense thick porridge, and an α-amylase-thinned calorie-dense porridge, were fed to stunted (*n* = 24) and healthy (*n* = 24) 18–30-month toddlers. Breath test results were expressed as Percent Dose Recovery and curve fit using the Weibull function to determine the kinetics of starch digestion.

**Results:**

The stunted and healthy toddlers were able to digest and oxidize the starch from traditional porridge equally well, with no statistically significant differences between the kinetic parameters of the two groups. After consumption of thickened porridge, healthy toddlers had slightly faster starch digestion kinetics with PDR curves rising more rapidly (*p* < 0.05) and peaking earlier in the postprandial period (*p* < 0.01) for healthy individuals than for stunted individuals, yet these groups did not have differences in the overall extent of starch digestion, as their final CPDR values were not significantly different. Gastric emptying rate did not differ significantly between the healthy and stunted groups.

**Conclusion:**

Overall, we found that thick porridge supplied digestible carbohydrates to stunted and healthy toddlers, as well as thinned calorie-dense porridge.

## Introduction

Digestion and absorption of macronutrients are essential for children’s growth and development, yet it is unclear whether stunted children can adequately digest and obtain energy from energy-dense porridges with thick texture. These foods are commonly part of the diet in sub-Saharan Africa. Stunting, defined as a child’s height that is at least two standard deviations below the average height for a child of the same age and sex [1], is a global health concern. Stunting is associated with increased risk for disease [2], poor educational outcomes [3], and economic disadvantages that persist into adulthood [4, 5]. In sub-Saharan Africa, the prevalence of stunting was estimated to be 39% of children under 5 years of age, and in Mali specifically it was estimated to affect 38% of children [6]. An increased understanding of food digestion and absorption among stunted children is important for the development of improved feeding strategies.

In sub-Saharan Africa, thin porridges fed to toddlers often have low energy density. For stunted toddlers, who lack adequate energy intake, it is desirable to increase the energy density of such foods without decreasing their digestibility [7]. Various approaches have been used to increase the energy density of porridges while maintaining thin consistency. These approaches include partial hydrolysis of starch through addition of commercial α-amylase [8], addition of malted grain [9], or using extrusion cooking [10].

Despite these efforts, the evidence is not conclusive that thinning porridges improves the ability of either stunted or healthy toddlers to increase energy consumption or to improve digestion. Bennett et al. [11] found that malnourished Peruvian children (*N* = 18, aged 8–17 months) had higher daily energy intake when presented with a low viscosity energy-dense porridge than when presented with a high viscosity energy-dense porridge or a low viscosity porridge of low energy density. Other studies have found no difference in energy intake between energy dense thick porridges and energy dense, enzymatically-thinned porridges fed to young children [12, 13]. It should be noted that these studies determined energy intake by multiplying the amount of food that was consumed by its energy density, which assumes that the starch from the porridges of varying energy density and texture was well digested. This assumption was most directly examined by Weaver et al. [14] using a ^13^C-breath test, who found that partial hydrolysis of starch using α**-**amylase in a maize-based complementary porridge increased the Cumulative Percent Dose Recovery (CPDR) of the ^13^C tracer, suggesting that the enzymatically thinned porridge was more digestible (*N* = 10, age 7–16 months). However, in that study only two of the ten participants were stunted, and therefore additional research is needed to clarify whether stunted toddlers can adequately digest starch from energy-dense thick porridges.

**Fig. 1:**
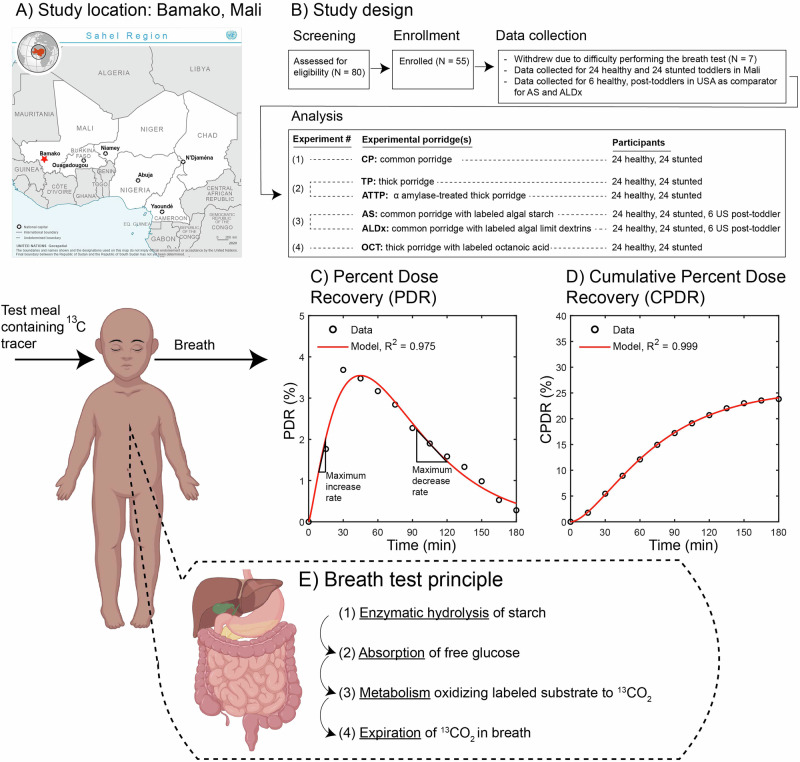
Study overview. The study was conducted in Bamako, Mali, shown with a red star. **A** on the United Nations Geospatial Services map of the Sahel region [44], where sorghum is a locally produced, climate resilient crop (Agnolucci et al. 2020). The study design is depicted in (**B**). Example PDR and CPDR curves are shown in (**C**) and (**D**), respectively. The physiological principle of the breath test is that starch must be digested and metabolized before the ^13^C tracer appears in the breath, with the steps in this process summarized in (**E**). The fraction of tracer labeled ^13^CO_2_ is proportional to digestion and metabolism of ingested target meal. Created using BioRender.

The aim of this study was to determine whether Malian stunted and healthy toddlers differ in their capacity to digest and metabolize starch from energy-dense thick and thin porridges. Figure 1 depicts our study design and introduces the ^13^C-breath test that was used to assess the digestion of the experimental porridges. Additionally, we sought to determine whether Malian stunted and healthy toddlers had differences in α-amylase sufficiency and gastric emptying rates, and whether these factors might impact overall starch digestion.

## Materials and methods

### Experimental overview and ^13^CO2 breath test experimental procedures

The study was performed in Bamako, Mali, at the Nutrition Center of the Centre Hospitalier et Universitaire Gabriel Toure. There was insufficient information to perform a power calculation due to our new applications of PDR and CPDR for assessing α-amylase sufficiency. However, based on previous work with the ^13^C-breath test (Cisse et al. [15]), we estimated 16 toddlers to be the minimum sample size needed. Considering potential dropouts, a total of 48 Malian toddlers (*n* = 24 healthy, *n* = 24 stunted) were recruited and participated in the study. They were aged 18-30 months (Table 1). A signed parent or caretaker informed consent form was obtained for each toddler. Ethical approvals were given by the National Ethical Committee for Health and Life Sciences in Mali and Purdue University (IRB #1209012695). The study was registered in clinicaltrials.gov (NCT03467737). Toddlers were scheduled for six consecutive days of testing. The experimental porridges were freshly prepared each day (Table 2). Feeding volumes were consistent at 200 mL of porridge and were completely consumed in the first 15 min of the test session. The thick porridges were spoon fed to the study participants, and the enzymatically-thinned porridge was consumed as a beverage. Although meal consumption can in principle affect postprandial metabolic outcomes [16], previous researchers found minimal effects when comparing an isoenergetic test meal consumed either in 5 min or in 30 min [17], and so in the present study we do not anticipate that differences in meal consumption rate would have affected outcomes, as all test meals were consumed in < 15 min. Each toddler fasted for at least 3 h before baseline breath collection. All breath samples were collected in aluminum lined bags (Cambridge Isotope; Andover, MA). The collected breath samples for each child and at each time point were analyzed for the presence of ^13^C in the expired CO_2_ using a non-dispersive infrared spectrophotometer (POCone, Otsuka Co, Japan) [15]. Samples were collected every 15 min for 3 h after the porridge was consumed.

**Table 1:** Characteristics of participants in the study, given as the mean ± standard deviation (standard error of the mean).

	Healthy	Stunted	US Children
*n*	24	24	6
Age (months)	25.3 ± 3.0 (0.61)	24.8 ± 3.5 (0.72)	49.0 ± 10.3 (4.21)
Weight (kg)	10.7 ± 1.0 (0.2)	9.6 ± 0.88 (0.18)	16.50 ± 3.8 (1.54)
Height (cm)	84.8 ± 3.53 (0.72)	79.2 ± 3.02 (0.62)	100.0 ± 6.8 (2.78)
HAZ	−0.85 ± 0.95 (0.193)	−2.50 ± 0.39 (0.08)	−0.79 ± 1.06 (0.431)

**Table 2:** Composition of experimental porridges.

	CP	TP	ATTP	AS	ALDx	OCT
	Common porridge with labeled sorghum flour	Thick porridge with labeled sorghum flour	Amylase treated thick porridge with labeled sorghum flour	Common porridge with labeled algal starch	Common porridge with labeled algal limit dextrins	Thick porridge with labeled octanoic acid
Sorghum flour (g)	16	20	20	16	16	20
Water (mL)	200	200	200	200	200	200
Beet sugar (g)	14	20	20	14	14	20
Waxy corn starch (g)	–	10	10	–	–	10
^13^C substrate (mg)	500 mg labeled sorghum flour	500 mg labeled sorghum flour	500 mg labeled sorghum flour	25 mg algal starch	25 mg algal limit dextrins	50 mg octanoic acid
Mixing time (min)	–	3	3	–	–	3
α-amylase (µL)	–	–	40	–	–	–
Energy density (kCal/g)	0.49	0.76	0.76	0.49	0.49	0.76
Energy density (kCal/mL)	0.57	0.95	0.95	0.57	0.57	0.95

A follow-up study on digestion of AS and ALDx in 6 older US children was conducted at the Baylor College of Medicine. Signed parent or caretaker consent forms were obtained and ethical approval was given by Baylor College of Medicine (IRB # H-37223). Experimental procedures were conducted in the same manner as in Mali.

### ^13^CO_2_ breath test data analysis

Percent Dose Recovery (PDR) and Cumulative Percent Dose Recovery (CPDR) were calculated from raw breath test data according to a previously described method [18]. CO_2_ production of each participant was estimated by multiplying a specific CO_2_ production constant [19, 20] by body surface area calculated using an established equation [21]. The calculation of PDR and CPDR thus accounts for differences in body weight through adjusting CO_2_ production of participants depending on their body mass. Additional information on the calculation of PDR and CPDR is provided in the Electronic Supplemental Information (Section 1.1).

Analysis of the kinetics of tracer recovery was carried out by fitting PDR and CPDR curves to Eq. 1 and Eq. 2, respectively [22].

1 $${PDR}(t)=a{t}^{b}\exp (-{ct})$$

2 $${CPDR}\left(t\right)=m{\left(1-\exp \left(-{kt}\right)\right)}^{\beta }$$

Where *t* is time (minutes), *a*, *b*, and *c* are constants in the PDR equation, and *m*, *k*, and *β* are constants in the CPDR equation. Curve fitting was conducted using MATLAB (R2022a, The Mathworks, Natick, MA) using the function *fit* (nonlinear least squares). The effect of each parameter on the shape of these curves was explored visually by changing one parameter at a time while holding the other two constant. This analysis is shown in the Electronic Supplemental Information section for PDR and CPDR (Supplemental Fig. 1).

Equation 1 and Eq. 2 provided a good fit to PDR and CPDR curves, respectively, with average R^2^ values across all runs of 0.978 for PDR and 0.999 for CPDR. Across the entire study, 301 breath test runs were recorded in total, and of these, four were identified as outliers due to poor curve fits to PDR and were excluded from analysis.

Additional parameters that were considered in this analysis were the maximum value of PDR (%), representing the greatest instantaneous rate of appearance of the ^13^C tracer, the time (min) corresponding to maximum PDR, the maximum increase rate (slope) of PDR (% min^−1^), the maximum decrease rate of PDR (% min^−1^) and the final value of CPDR (%), representing the cumulative percentage of ^13^C tracer recovered during the breath test.

### ^13^CO_2_ breath test materials

Experimental porridges were made with a mixture of unlabeled sorghum flour, ^13^C-labeled sorghum flour, and other components as listed in Table 2. The ^13^C substrates were ^13^C-labeled sorghum flour, ^13^C-algal starch, ^13^C-algal limit dextrin, and ^13^C-octanoic acid. The experimental sorghum porridges were common porridge (CP); starch-augmented, energy dense thick porridge (TP); starch-augmented, energy dense, α-amylase thinned porridge (ATTP); common porridge with ^13^C-labeled algal starch (AS); common porridge with ^13^C-labeled algal starch hydrolysates (ALDx); and starch-augmented, energy dense porridge with ^13^C-octanoic acid (OCT). The use of ^13^CO_2_ breath tests for analyzing starch digestion kinetics by combining enriched substrates produced through photosynthetic fixation of ^13^C with non-labeled substrates was pioneered and validated by previous researchers [23, 24].

All porridges were prepared on the test day following the typical preparation procedures for weaning foods in Mali and fed to participants at approximately 37 °C. A slurry was made of sorghum flour and 1/3 of total water (200 mL) and then added into the remaining 2/3 water at a boiling condition. The mixture was cooked for 6 min. Lemon juice and sugar were added for taste. Sugar from beets, which are a C3 plant naturally with lower ^13^C signals than C4 plants (ex. sugar cane), was selected for this study.

The chronology of ^13^C-labeled breath test feeding experiments was: AS, ALDx, CP, TP, ATTP and OCT. Due to the logistical constraint of being able to prepare only one meal per day in the clinic, all toddlers received the same test meal on each day. Since the length of each test was 3 h, to avoid hunger, an unlabeled sweetened rice porridge was served 90 min after test ingestion. The AS and ALDx breath test experiments were repeated with a group of six older US children (40-56 months) so that α-amylase sufficiency of the Malian groups (healthy and stunted toddlers) could be compared with US children.

### Unlabeled and labeled sorghum flours in CP, TP, and ATTP breath test experiments

Unlabeled sorghum porridge was made with a Malian sorghum variety “Darrel Ken”, derived from the crossing of an improved Guinea-type variety (N’Tenimissa) and a local Guinea type variety (Seguetana) for grain quality and yield. Processing of the unlabeled sorghum was decorticated in an abrasive dehuller and milled to flour using a hammer-mill. The ^13^C-labeled sorghum flours used in CP, TP, and ATTP were sorghum variety CSC3XR28 – F1 hybrid grown and greenhouse-enriched with ^13^CO_2_ at the USDA-ARS Children’s Nutrition Research Center at Baylor College of Medicine (Houston, TX) [25]. Labeled sorghum grains were decorticated using a tangential abrasive dehulling device (TADD, Venables Machine Works, Ltd, Saskatoon, Canada) for 3 min and ground into flours with a coffee grinder. Starch content in sorghum flours was quantified using the Total Starch Assay Kit (Megazyme, Wicklow, Ireland). A food-grade thermostable α-amylase was obtained from DuPont Industrial Biosciences (Wilmington, DE). Enrichment of ^13^C-glucose in the sorghum starch was carried out by hydrolysis to free glucose, and measurement by gas chromatography combustion isotope ratio mass spectrometry (GC/C/IRMS) using penta-acetate derivatives [25]. The ^13^C enrichment of labeled sorghum (153.71 ± 0.958 APE ‰) was ca. 4,500 times higher than unlabeled sorghum (0.033 ± 0.551 APE ‰, *p* < 0.001) and ca. 1500 times higher than maize (0.105 5. ± 0.004 APE ‰, *p* < 0.001). Exact enrichment values are shown in Supplemental Table 2. No differences would be expected in starch digestibility between the local sorghum used to prepare the unlabeled porridge and the ^13^C-enriched sorghum, as differences in the digestibility of cooked starches were found to be similar in different sorghum cultivars [26].

### Other ^13^C substrates in AS, ALDx, and OCT breath test experiments

^13^C-labeled algal starch and ^13^C-octanoic acid were purchased from Sigma-Aldrich (St. Louis, MO). ^13^C-algal starch hydrolysates (ALDx), containing α-oligomers and limit dextrins, was prepared through hydrolysis with α-amylase at pH 6.9 at 37 °C for 2 h, at which point the hydrolysis product (reducing sugar) concentration did not have a significant change.

### Viscosity of CP, TP, and ATTP

The viscosity of the sorghum porridges was determined by flow curve measurements using a stress-controlled rheometer (AR-G2 Rheometer, TA Instruments, New Castle, DE). Measurements were made using a cone-plate assembly. A small porridge sample was loaded onto the plate and the viscosity was determined up to a shear rate of 300 s^−1^ at 37 °C. Analyses were performed in duplicate.

### Maturation of α-amylase activity (AS/ALDx ratio)

During digestion, starch must first be hydrolyzed by luminal α-amylases and then by brush-border α-glucosidases before absorption of ^13^C-labeled glucose can occur. This principle was leveraged to design a test for sufficiency of α-amylases, by comparing the appearance of tracer in the breath after consumption of common porridge with labeled algal starch (AS) or labeled algal limit dextrins (ALDx). Intact algal ^13^C-starch must first be hydrolyzed by α-amylase followed by hydrolysis by mucosal α-glucosidases before the tracer appears in the breath. On the other hand, pre-hydrolyzed algal ^13^C-starch (ALDx) effectively bypasses α-amylases and therefore appearance of the tracer is limited only by the mucosal α-glucosidases. Thus, the appearance of ^13^CO_2_ in the breath following consumption of AS relative to ALDx provides an indication of the sufficiency of α-amylases in the digestion of starch. For example, a greater concentration of ^13^CO_2_ after consuming ALDx relative to AS indicates that α-amylase activity was rate limiting in the overall process of digestion, absorption, and metabolism. The final value of CPDR represents the total ^13^C that appeared in the breath as a percentage of the amount that was initially present in the porridge, therefore we defined the α-amylase sufficiency ratio as the final value of CPDR from AS divided by the final value of CPDR from ALDx. A value of 1.0 indicates that the recovery of ^13^C was equivalent between the two porridges, implying that α-amylase activity was not rate limiting. Values < 1.0 indicate that a greater amount of ^13^C was recovered from the pre-digested algal starch, indicating insufficiency of α-amylase activity.

### Gastric emptying of 13C-octanoic acid (OCT)

The objective of the OCT breath test experiment was to determine whether gastric emptying of an energy-enriched thick sorghum porridge (TP) was reduced in stunted toddlers. The principle of the test is that ^13^C-octanoic acid is resistant to gastric conditions, but rapidly absorbed and oxidized in the small intestine [27, 28]. ^13^C-octanoic acid (50 mg) was added to the TP to obtain a test porridge for assessment of gastric emptying (OCT). PDR and CPDR were determined and modeled as previously described. Gastric emptying half time, lag time, and gastric emptying coefficient were calculated using Eqs. 3, 4, and 5, respectively [22].

3 $${T}_{1/2}=-1/{\rm{kln}}(1-{2}^{-\frac{1}{\beta }})$$

4 $${T}_{{lag}}=\mathrm{ln}\left(\beta \right)/k$$

5 $${GEC}=\mathrm{ln}(a)$$

Where ${T}_{1/2}$ (min) is the gastric emptying half time, ${T}_{{lag}}$ (min) is the gastric emptying lag time, ${GEC}$ (unitless) is the gastric emptying coefficient, *k* and *β* are constants from Eq. 2, and *a* is a constant from Eq. 1.

### Statistical analysis

Results from the CP experiment were analyzed using one-factor ANOVA to determine the influence of participant stunting status (stunted, healthy) on the parameters from the breath test (PDR parameters: *a*, *b*, *c*; CPDR parameters: *m*, *k*, *β*), and other values from PDR and CPDR analysis (maximum value of PDR, time corresponding to maximum PDR, maximum increase and decrease rates of PDR, and final value of CPDR). Analysis using two-factor ANOVA with participant stunting status and sex (M, F) was also carried out.

Data from the experiment with TP and ATTP were analyzed using two-factor analysis of variance to determine the influence of participant status (healthy, stunted), porridge type (TP, ATTP), and their interaction on the parameters listed above. As before, follow-up analysis was carried out by fitting three-factor ANOVA models with participant stunting status, porridge type, and participant sex as factors.

Data from the AS and ALDx experiment were analyzed using two-factor ANOVA to determine the influence of participant status (healthy, stunted, US children), porridge type (AS, ALDx), and their interaction on the parameters from the breath test, with follow-up analysis using three-factor ANOVA that also included participant sex as a factor. The α-amylase sufficiency ratio was quantified as the final value of CPDR from the ALDx experiment divided by the final value of CPDR from the AS experiment (Section 3.8) and was analyzed using one-factor ANOVA with participant status (stunted, healthy) as the factor.

Gastric emptying parameters (${T}_{1/2},\,{T}_{{lag}}$ and ${GEC}$) were analyzed using one-factor ANOVA with participant status (healthy, stunted).

For all statistical models, homogeneity of variance of the model residuals was assessed using the Brown-Forsythe test and normality was assessed using the Anderson-Darling test. If either test was not satisfied (*p* < 0.05) then a nonparametric analysis was performed using aligned rank-transform followed by analysis of variance [29, 30].

Significance levels of all factors in all ANOVA models are provided in Supplemental Tables 7-10.

## Results

We first conducted an experiment to determine whether stunted and healthy toddlers differed in their ability to digest a common sorghum porridge (CP, control). To examine whether thick and thin porridges differed in their digestion profile, CP was augmented with additional starch, resulting in a thick, energy-dense porridge (TP). Augmentation with starch and treatment with α-amylase was used to create an energy-dense, thin porridge (ATTP). We hypothesized that stunted toddlers, who would more likely be α-amylase insufficient, would digest the thick porridge (TP) less well than the healthy toddlers, but would not differ in their capacity to digest energy-dense, α-amylase thinned porridge (ATTP). Each meal consumed by study participants (CP, TP, and ATTP) contained 500 mg of ^13^C-enriched sorghum flour for breath test analysis.

To determine the sufficiency of α-amylase with respect to the overall digestion of starch, we compared the digestion of a common sorghum porridge with ^13^C-labeled algal starch (AS) and common sorghum porridge with ^13^C-labeled algal limit dextrins (ALDx). Since the labeled starch in ALDx was predigested by α-amylase, comparing the recovery of ^13^C between AS and ALDx was used to determine whether study participants had sufficient α-amylase activity. Gastric emptying was quantified using the porridge with additional starch and ^13^C-labeled octanoic acid (OCT). The composition of the porridges is given in Table 2.

**Fig. 2:**
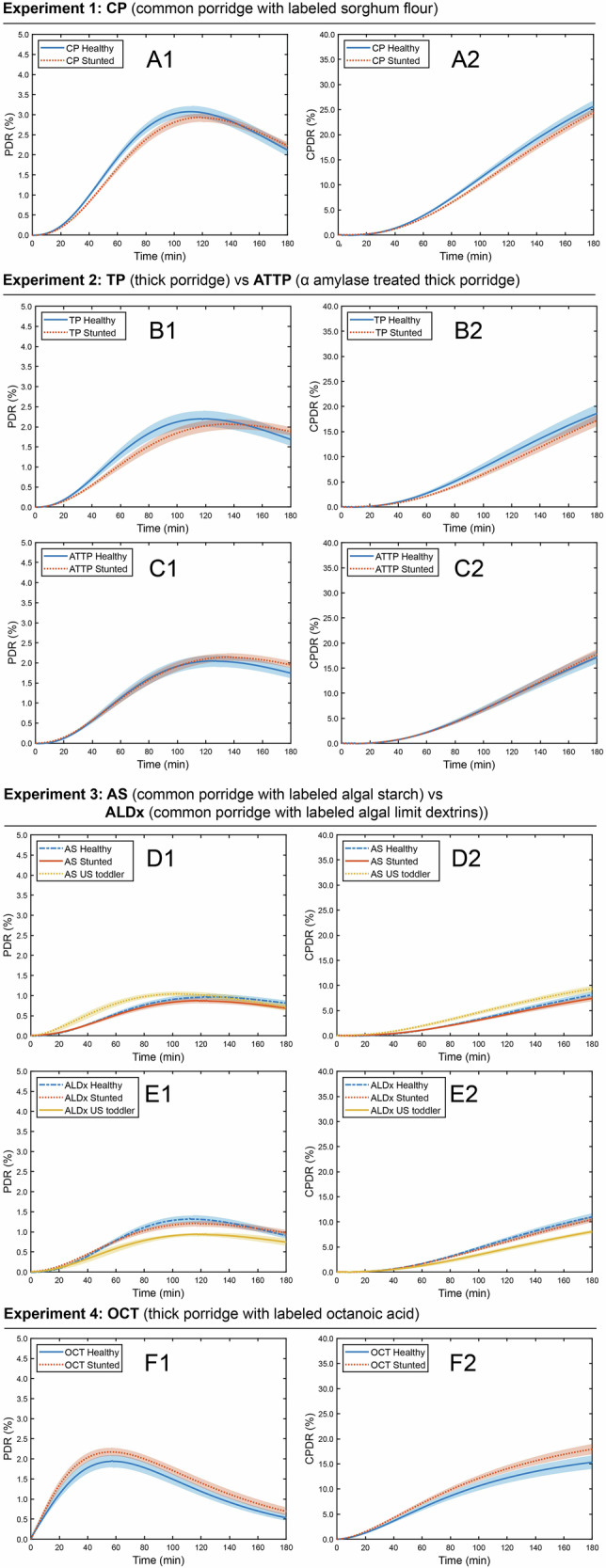
Breath test curves showing PDR (left column, A1-F1) and CPDR (right column, A2-F2) for the experimental meals. The mean breath test response is shown with a line and the standard error of the mean is represented by the shaded region around the line. The average and standard error of the mean for each response were obtained by evaluating all the functions for PDR or CPDR (obtained from curve fitting) for a given treatment, and then taking the average response at each timepoint along with the standard error.

Characteristics of participants in the study are shown in Table 1. All the breath test curves analyzed in this study are provided in the Supplemental Information Section (Supplemental Fig. 3–16) with the four outliers identified in red boxes. Average PDR and CPDR breath test curves for each porridge are shown in Fig. 2. Parameters from the curve fits are given in Supplemental Tables 3–6. The significance level of the factors in the study (participant stunting status, porridge type, and participant sex) with regards to each parameter in the analysis are given in Supplemental Tables 7–10.

### Effect of stunting status on digestion of common sorghum porridge (CP)

Regardless of stunting status, Malian toddlers digested and oxidized starch from CP with similar kinetics, as evidenced by a lack of significant differences in any parameter from PDR and CPDR analyses. Average breath test curves for CP are shown in Fig. 2 for PDR (A1) and for CPDR (A2). The parameters of PDR and CPDR curves from this experiment are given in Supplemental Table 3, are visualized in Supplemental Fig. 2, and the significance levels of main effects are provided in Supplemental Table 7.

A follow-up analysis was conducted using two-factor ANOVA with both participant status (stunted, healthy) and participant sex (male, female) as the factors, and it was determined that males had a significantly (*p* < 0.05) higher peak value of PDR as compared to females (3.36 ± 0.48% vs. 2.86 ± 0.58%). The peak of PDR occurred earlier in the postprandial period for males as compared to females (115.5 ± 14.2 min vs. 122.7 ± 22.4 min, respectively, *p* < 0.05), and had a significantly greater maximum increase rate as well as a greater maximum decrease rate. Analysis of the endpoint value of CPDR showed that a significantly (*p* < 0.05) greater total percent of the tracer was recovered for males (26.9 ± 4%) as compared to females (23.4 ± 4.8%).

### Effect of porridge viscosity

An energy-dense, thick porridge (TP) was created by augmenting the common porridge (CP) with waxy corn starch and additional sorghum flour (Table 2). α-Amylase treatment of TP yielded an isoenergetic, enzymatically thinned porridge (ATTP), which was compared with TP to determine the effect of porridge rheology on the kinetics of starch digestion. α-Amylase treatment resulted in a lower viscosity of ATTP as compared to TP. CP and TP had initial viscosities in the range of 200-300 Pa-s which is similar to that of oatmeal, while ATTP was < 100 Pa-s with a more liquid-like texture. The thick porridges were spoon fed to the study participants, and the ATTP was consumed as a beverage. At a shear rate of 50 s^−1^, which is considered to resemble the conditions of swallowing (Stokes et al., 2013; Dirven et al., 2017), the viscosity values reduced to 1.6 Pa-s, 2.9 Pa-s, and 0.17 Pa-s for CP, TP, and ATTP, respectively, showing the large enzyme thinning effect.

**Fig. 3:**
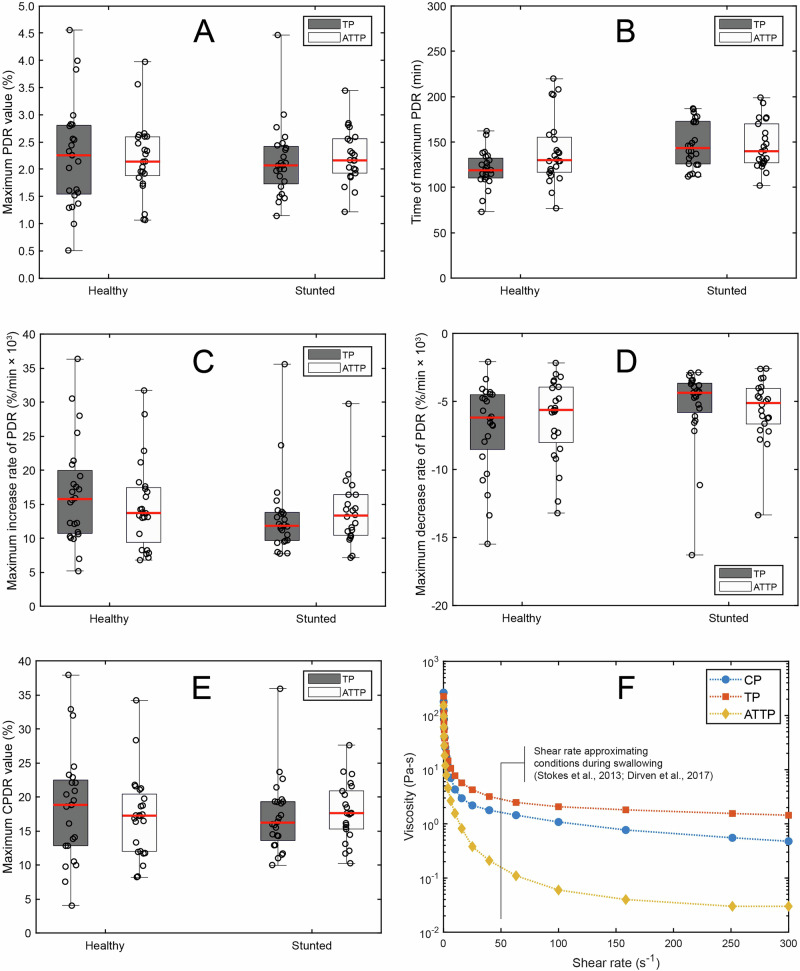
Energy-dense, thick and enzymatically thinned porridges showed no difference in starch digestion. Parameters (**A**–**E**) from breath test analysis for the experiment comparing digestion of TP and ATTP in healthy Malian toddlers and stunted Malian toddlers. Raw values are shown as black circles. The box extends from the 25^th^ to the 75^th^ percentile, with the whisker extending from the lowest to the highest observed value. The red horizontal line denotes the median observation. Viscosity by shear rate of the three porridges used in the study is in (**F**).

Porridge type (TP vs. ATTP) did not significantly influence any outcome variable from the breath test (Fig. 3, Supplemental Table 8) and similarly the interaction effect between porridge type (thick vs thin) and participant stunting status was not significant. Notably, maximum values of PDR (Fig. 3A) and CPDR (Fig. 3D) were not influenced by participant stunting status. This shows that reducing porridge viscosity using α-amylase had no impact on the overall extent of starch digestion in either stunted or healthy toddlers.

Average breath test curves for these porridges are shown in Fig. 2 for PDR (B1, C1) and for CPDR (B2, C2). Parameter values from PDR and CPDR analysis are shown in Supplemental Table 4 with significance levels of main effects and interactions given in Supplemental Table 8. It was found that participant sex significantly (*p* < 0.05) influenced every parameter that was studied except for the *m* parameter from the CPDR curve fits and so models including participant sex were used in the analysis of this experiment.

We quantified subtle differences in the kinetics of starch digestion between healthy and stunted individuals. Specifically, PDR curves rose more rapidly (*p* < 0.05, Fig. 3C), peaked earlier in the postprandial period (Fig. 3B, *p* < 0.01) and then declined more rapidly (Fig. 3D, *p* < 0.05) for healthy individuals than for stunted individuals. These differences were driven by the thick porridge (TP). When the data from only ATTP were analyzed, there were no longer any differences between stunted and healthy toddlers in these minor kinetic parameters.

As with the CP experiment, males had a significantly (*p* < 0.05) higher peak value of PDR as compared to females (2.44 ± 0.79% vs. 2.04 ± 0.61%), that occurred significantly (*p* < 0.05) earlier in the postprandial period (127.4 ± 24.2 min vs. 145.8 ± 30.6 min), was marked by a more rapid increase as well as a more rapid decrease in PDR, and led to a significantly (*p* < 0.05) greater endpoint value of CPDR (19.4 ± 6.9% vs. 16.3 ± 5.1%).

### Sufficiency of α-amylase activity

The common sorghum porridge with ^13^C-labeled algal starch (AS) was compared to the common sorghum porridge with ^13^C-labeled algal limit dextrins (ALDx) to determine the sufficiency of α-amylase activity in three different groups: healthy Malian toddlers, stunted Malian toddlers, and children in the United States (US) as a comparator. Overall, we found that Malian toddlers exhibited α-amylase insufficiency, which was not displayed by the US children. PDR and CPDR curves after consumption of AS and ALDx are shown in Fig. 2. Parameter values from PDR and CPDR functions are given in Supplemental Table 5 and significance levels of main effects and interactions are given in Supplemental Table 9. Participant sex significantly influenced only one parameter (the maximum decrease rate of PDR) out of the 11 parameters that were quantified, and therefore models including only participant status and porridge type were used for analysis of this experiment.

**Fig. 4:**
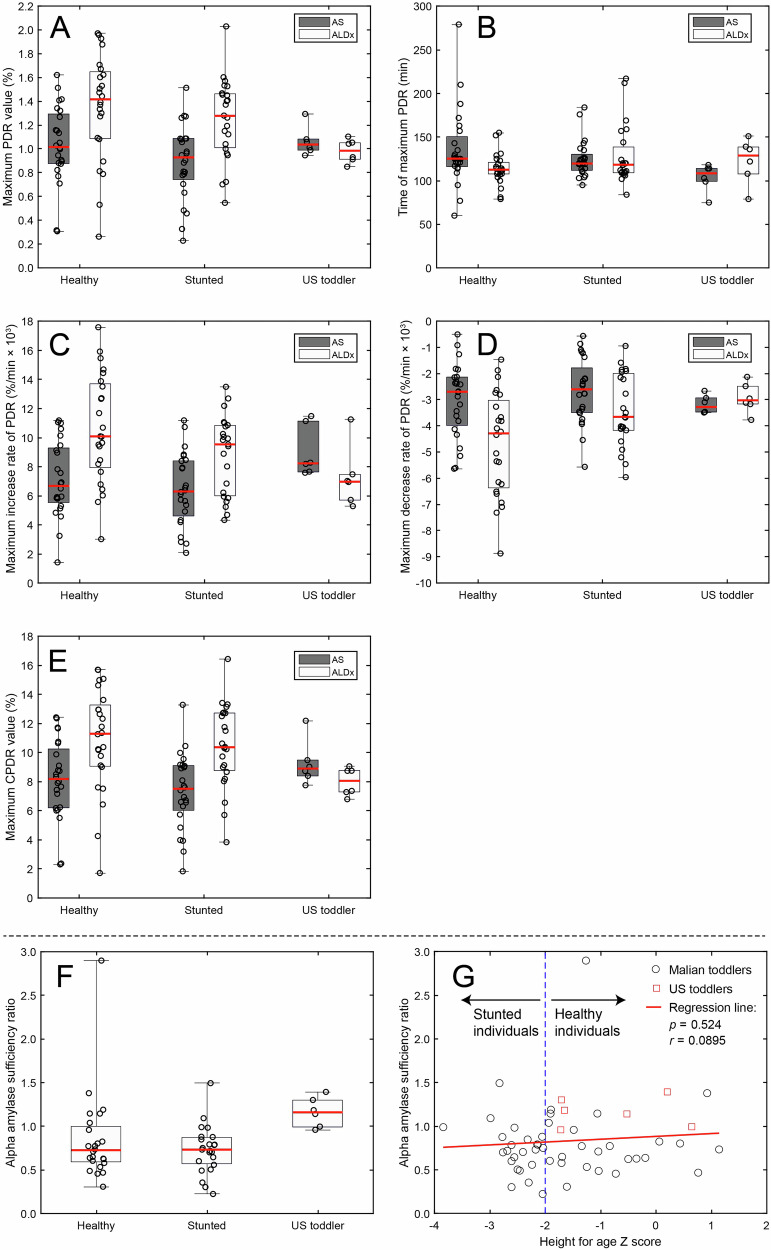
Parameters from breath test analysis for the experiment comparing digestion of AS and ALDx in healthy Malian toddlers, stunted Malian toddlers, and US children. **A**–**E** Raw values are shown as black circles. α-Amylase sufficiency ratio for healthy Malian toddlers, stunted Malian toddlers, and US is (**F**). For all box plots (**A**–**F**), raw values are shown as black circles, the box extends from the 25^th^ to the 75^th^ percentile, the whisker extends from the lowest to the highest observed value, and the red horizontal line denotes the median observation. Regression of α-amylase sufficiency ratio onto height-for-age Z-score (**G**). No significant relationship was observed.

The maximum value of PDR (Fig. 4A) was significantly influenced by porridge type (*p* < 0.001), participant status (*p* < 0.05), and their interaction (*p* < 0.05). Maximum PDR was greater for ALDx than for AS (1.30 ± 0.40% vs. 0.96 ± 0.33%, respectively) but importantly, only in Malian toddlers. In US children, there was no difference between the maximum value of PDR from ALDx and AS. This shows that Malian toddlers (both stunted and healthy) had a higher peak level of ^13^C in the breath after consuming porridge with labeled algal limit dextrins as compared to labeled algal starch, indicating some degree of α-amylase insufficiency that was not observed in the US children.

The time of maximum PDR (time of the peak, Fig. 4B) was not significantly influenced by porridge type or participant status. The maximum increase rate of PDR (Fig. 4C) and the maximum decrease rate of PDR (Fig. 4D) were significantly influenced by porridge type (*p* < 0.05) but not by participant status, with both a significantly greater increase rate (*p* < 0.05) and a significantly greater decrease rate (*p* < 0.01) for ALDx as compared to AS. Similarly, the maximum value of CPDR, representing the overall recovery of the ^13^C tracer in the breath, was significantly influenced by porridge type (*p* < 0.05), but not by participant status. There was greater overall recovery of the ^13^C tracer from ALDx as compared to AS in Malian toddlers, with maximum CPDR values of 10.6 ± 3.3% vs. 7.7 ± 2.7%, respectively, an effect that was not present in US children where recovery of the tracer from ALDx and AS did not differ. Robayo-Torres et al. [31] showed progressively lower ^13^C-CO_2_ recovered in breath as age of children increased, which is in agreement with the lower value of CPDR for the older US cohort after consumption of the predigested ALDx. Yet, the US children had similar values of CPDR for AS as the Malian toddlers, thus giving a higher relative α-amylase sufficiency ratio.

The α-amylase sufficiency ratio was defined as the final value of CPDR from AS divided by the final value of CPDR from ALDx (Section 3.8, Fig. 4F). A value of 1 would indicate an equivalent recovery of ^13^C in the breath from both porridges, and thus that α-amylase activity was sufficient. The α-amylase sufficiency ratio was not significantly different between stunted and healthy Malian toddlers but was significantly (*p* < 0.05) greater for US children than for either stunted or healthy Malian toddlers (Fig. 4E), again showing that there was some degree of α-amylase insufficiency in the Malian toddlers regardless of stunting status. Follow-up analysis was conducted by regression of the α-amylase sufficiency ratio onto height-for-age Z score (HAZ) and the relationship was not significant (*p* = 0.52, Fig. 4G). Furthermore, α-amylase sufficiency ratio was not significantly influenced by participant sex. In summary, we found that α-amylase insufficiency was present in Malian toddlers regardless of stunting status or sex, whereas US children did not have α-amylase insufficiency.

### Gastric emptying

Gastric emptying was examined using the octanoic acid breath test in another experiment conducted in the healthy and stunted Malian toddler groups. Breath test curves from the octanoic acid breath test (OCT, Figs. 2F1 and F2) were analyzed to determine whether stunted and healthy toddlers exhibited differences in gastric emptying rate (see Methods).

**Fig. 5:**
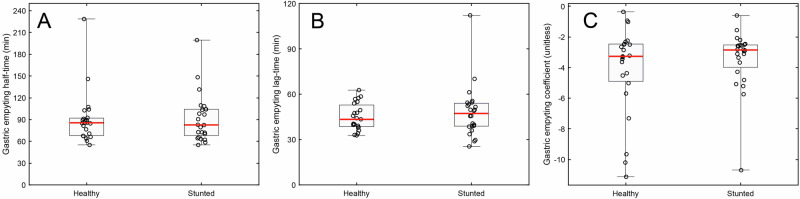
Gastric emptying rates did not differ between healthy and stunted toddlers. Gastric emptying half-time (**A**), lag time (**B**), and gastric emptying coefficient (**C**) for healthy Malian toddlers and stunted Malian toddlers. Raw values are shown as black circles. The box extends from the 25^th^ to the 75^th^ percentile, with the whisker extending from the lowest to the highest observed value. The red horizontal line denotes the median observation.

Participant stunting status (stunted, healthy) did not significantly influence gastric emptying half-time, gastric emptying lag-time, or gastric emptying coefficient (Fig. 5), indicating that stunted toddlers did not have a different gastric emptying rate as compared to healthy toddlers. None of the gastric emptying parameters were influenced by participant sex.

## Discussion

Key findings from this study indicate that stunted toddlers digested and obtained energy from energy dense, thick porridge (TP) equally well as from energy dense enzymatically thinned porridge (ATTP). This was counter to our original hypothesis that the stunted toddlers would digest the energy-dense thin porridge (ATTP) better than the energy dense thick porridge (TP). Furthermore, stunted and healthy toddlers digested and obtained energy from TP and ATTP equally well. Previous studies looking at energy intake of porridges of varying energy density and texture have assumed that starch, even from energy-dense thick porridges, is well digested by toddlers, regardless of their nutritional status. Here, we show stunted toddlers digest and obtain energy from energy-dense thick porridges. This supports previous reports that energy dense porridges led to greater energy intake in children as compared to low energy density porridges [11–13, 32, 33]. Yet, an important distinction is that these studies assumed that starch from energy dense porridges, even those with thick texture, would be well digested. Our results show that this was true for both stunted and healthy toddlers in Mali. Stunted children require a great deal of energy for catch up growth [34], and therefore identifying digestible, energy dense foods for them is a priority.

Treatment of porridges with α-amylase has been used to increase the energy and nutrient density of the diet in cultural settings where young children are traditionally fed liquid foods. In our study the energy-dense, thick porridge (TP) was treated with α-amylase to create the isoenergetic, enzymatically thinned porridge (ATTP). Despite finding that the overall digestion of TP and ATTP did not differ in either the stunted or healthy cohorts (as indicated by the Cumulative Percent Dose Recovery, CPDR), we nevertheless identified subtle differences in the kinetics of starch digestion due to participant stunting status and porridge texture. For TP, Percent Dose Recovery (PDR) curves for healthy individuals rose more quickly, peaked earlier in the postprandial period, and then declined more quickly than for stunted individuals. These minor differences were only present in the TP and disappeared for the ATTP. These differences are predicted to be of low clinical significance since the maximum value of PDR, representing the highest instantaneous recovery rate of tracer, as well as the maximum value of CPDR, representing the cumulative recovery of tracer, were not significantly affected by participant stunting status. In other words, the tracer recovery curves had slight differences in shape between the stunted and healthy toddlers after consumption of TP, but at the conclusion of the testing period these curves represented the same overall extent of starch digestion.

The most direct published report to compare our findings is that of Weaver et al. [14] who used a ^13^C-breath test to find that treating a maize-based complementary porridge with α**-**amylase increased CPDR of the ^13^C tracer, showing that the enzymatically thinned porridge was more digestible. In our study, the final value of CPDR from TP was 18.6 ± 8.2% for the healthy individuals and 17.1 ± 5.4% for the stunted individuals. For ATTP, the final values were 17.1 ± 6.0% and 17.8 ± 4.3%, respectively.

Our original hypothesis was that stunted toddlers would have impaired ability to digest starch from thick porridges. This was based on traditional thinking that porridges should be thinned for stunted children, but also on research showing that moderately malnourished wasted or stunted children have impaired ability to digest starch due to pancreatic α-amylase deficiency. Since the 1950’s research has shown that malnutrition causes insufficiency of pancreatic enzyme production, including α-amylase that digests starch [35]. More recent evidence suggests a similar problem due to moderate malnutrition. In Colombia, Watson et al. [36] found marginal malnutrition suppressed production of both pancreatic and salivary amylases in children (mean age 21 months). Sauniere and Sarles [37] showed pancreatic insufficiency with markedly lower amylase activity in duodenal aspirations in Abidjan children with kwashiorkor (73.9 U·ml^−1^) compared to control African children (186.9 U·ml^−1^) and French children (263.5 U·ml^−1^), and in Dakar clinically malnourished and control (though moderately malnourished) weaned children both showed low amylase activity of 50.2 and 109.7 U·ml^−1^. In Zambia, lower sucrase isomaltase expression has been noted in stunted toddlers as compared to adults [38]. Despite these differences in enzyme activity, it should be noted that the activity of a single type of enzyme measured in digestion fluid does not describe the overall capacity for digestion in the individual [39]. In the present study, the use of the ^13^C-breath test allowed us to study the overall capacity for starch digestion.

To analyze the sufficiency of endogenous α-amylase, we tested a porridge with ^13^C-labeled algal starch (AS) and a porridge with ^13^C-labeled algal limit dextrins (ALDx). By comparing the cumulative recovery of ^13^C in the breath between the two porridges, we found that Malian toddlers, both stunted and healthy, exhibited α-amylase insufficiency, which was not observed in the US children (Fig. 4F). It should be noted that the US children were an average of 4 years old – roughly 2 years older than the Malian toddlers (Table 1). Salivary α-amylase has mature activity at ca. 3 months of age [40], whereas pancreatic α-amylase activity can be detected at 6 weeks of age but remains low until ca. 6 months [41] during which period exogeneous α-amylases present in breast milk may partially compensate. The function of α-amylase is important because starch polymers must be broken down to absorbable free glucose before absorption and use as an energy source. The digestion of starch begins with hydrolysis by salivary α-amylase in the mouth, followed by hydrolysis by pancreatic α-amylase in the lumen of the small intestine. Hydrolysis by α-amylase results in smaller oligosaccharides of linear and branched α-limit dextrins which are the substrates for four mucosal (brush border) α-glucosidases, expressed by two proteins, maltase-glucoamylase and sucrase-isomaltase [42]. Free glucose resulting from hydrolytic action of these enzymes can then be absorbed. Previous research has shown that mucosal α-glucosidases do not only convert post-α-amylase limit dextrins to glucose, but can also participate in the primary hydrolysis of starch [43], thus potentially compensating for the α-amylase insufficiency in Malian toddlers that was identified in this study.

In this study, we report quantitative analyses of the kinetics of starch digestion from complementary porridges in stunted and healthy toddlers in Mali. Overall, we found that stunted toddlers digested starch from thick and thin energy-dense porridges equally well, and that they digested the porridges as well as healthy toddlers.

## Supplementary information

Supplementary Information

## Data Availability

Breath test data collected in this study are available in the figshare repository located at: 10.6084/m9.figshare.28281185.v1.
