# Supplementary material for: Stunted African toddlers digest and obtain energy from energy-dense thick sorghum porridge
Source: Eur J Clin Nutr. 2025 May 23;79(10):1018–28. doi: 10.1038/s41430-025-01632-y (PMC12537497; doi:10.1038/s41430-025-01632-y)
Supplement: Supplementary file 1 — Supplementary Information [file 41430_2025_1632_MOESM1_ESM.docx]

**Electronic supplemental information for:**

**Stunted African toddlers digest and obtain energy from energy-dense thick sorghum porridge**

Fatimata Cisse ^a,b,†^, Clay Swackhamer ^a,c,†^, Hawa G. Diall ^d^, Atossa Rahmanifar ^e^, Mariam Sylla ^d^,

Antone R. Opekun ^f^, Michael A. Grusak ^g,h^, Amy H-M. Lin ^a,i^, Elizabeth A. Pletsch ^a,j^, Anna MR. Hayes ^a,k^, Roberto Quezada-Calvillo ^g,l^, Buford L. Nichols ^f^, Bruce R. Hamaker ^a,*^

^a^ Whistler Center for Carbohydrate Research, Department of Food Science, Purdue University, West Lafayette, IN, 47906, USA

^b^ Institut d’Economie Rurale du Mali (IER), BP 258, Bamako, Mali

^c^ Present address: Nutrition Program, School of Nutrition and Public Health; Department of Food Science and Technology; Oregon State University, Corvallis, OR, 97333, USA

^d^ Département de Pédiatrie, Centre Hospitalier et Universitaire Gabriel Toure, BP 267, Bamako, Mali

^e^ Nutrition Consultant, West Lafayette, IN, 47906, USA

^f^ Departments of Medicine & Pediatric Sections of Gastroenterology and G.I., Baylor College of Medicine, Houston, TX, 77030, USA

^g^ USDA-ARS Children’s Nutrition Research Center, Houston, TX, 77030-2600

^h^ Present address: USDA-ARS Edward T. Schafer Agricultural Research Center, Fargo, ND, 58102, USA

^i^ Present address: Singapore Institute of Food and Biotechnology Innovation, Agency for Science, Technology and Research, Singapore

^j^ Present address: USDA-ARS Beltsville Human Nutrition Research Center, Beltsville, MD, USA

^k^ Present address: Department of Food Science and Technology, Oregon State University, Corvallis, OR, 97333, USA

^l^ Facultad de Ciencias Químicas, Universidad Autónoma de San Luis Potosí, Zona Universitaria, San Luis Potosí, 78210, Mexico

^†^ indicates co-first authors

^*^ indicates corresponding author

# Materials and methods

## ^13^CO_2_ breath test data analysis

Percent Dose Recovery (PDR) of ^13^C in the breath test was calculated according to the method of Amarri et. al., (1995):

| $PDR_{from t to t_{+1}} =\frac{\left[ \left( \delta_{t}^{13}-\delta_{0}^{13} \right)+\left( \delta_{t+1}^{13}-\delta_{0}^{13} \right) \right]/2 \times{(t}_{+1}-t)\times R_{PDB}\times{10}^{-3}\times C}{\frac{mg substrate}{molecular weight} \times\frac{P\times n}{100}}$ | (1) |
| --- | --- |

Where $\left( \delta_{t}^{13}-\delta_{0}^{13} \right)$ represents the abundance of ^13^C in a breath sample relative to an ambient air reference sample at time $t$, $\left( \delta_{t+1}^{13}-\delta_{0}^{13} \right)$ represents the same quantity but at time $t_{+1}$, ${(t}_{+1}-t)$ is the time interval (15 min), $R_{PDB}$ is the isotopic abundance of ^13^C in PeeDee Belemnite, an international standard material ($R_{PDB}=0.0112372)$, $C$ is the estimated production rate of CO_2_ by the participant, $mg substrate$ is the mass of the ^13^C**-**labeled substrate consumed as part of the test porridge, $molecular weight$ is the molecular weight of the substrate, $P$is the atom % excess of ^13^C in the substrate, and $n$ is the average number of ^13^C labelled carbons per molecule of substrate. For our system (POCone, Otsuka Co, Japan), $\left( \delta_{t}^{13}-\delta_{0}^{13} \right)$is reported by the instrument as ^13^CO_2_ delta over baseline.

CO_2_ production rate was conducted for each participant according to the method of Shreeve et. al., (1970):

| $CO_{2} production rate =300 mMol CO_{2}/ h\times SA$ | (2) |
| --- | --- |

Where $SA$ is the participant’s body surface area, estimated using the method of Haycock et. al., (1978):

| $SA =0.024265 \times w^{0.5378}\times h^{0.3964}$ | (3) |
| --- | --- |

Where *w* is participant weight (kg) and *h* is participant height (cm) which were measured at the study center. Parameter values used for the calculation of PDR were adjusted based on the form of the ^13^C in each experimental porridge (Supplemental Table 1):

**Supplemental Table 1.** Constants used in the calculation of PDR and CPDR for the test porridges.

| **Experiment** | $\boldsymbol{Mg substrate}$ **(mg)** | $\boldsymbol{Molecular weight}$ **(g/Mol)** | $\boldsymbol{P}$ **(%)** | ***n*** |
| --- | --- | --- | --- | --- |
| AS vs ALDx | 25 | 168.14 | 99 | 6 |
| CP | 500 | 168.14 | 4.66 | 6 |
| TP vs ATTP | 500 | 168.14 | 4.66 | 6 |
| OCT | 50 | 145.2 | 99 | 1 |

**Supplemental Table 2.** ^13^C enrichment in glucose from various flours and starches.

|  | **Number of replicates** | **APE (%_0_)** |
| --- | --- | --- |
| Unlabeled sorghum | 3 | 0.033 ± 0.551 |
| Labeled sorghum | 8 | 153.71 ± 0.958 |
| Maize starch | 3 | 0.105 ± 0.004 |
| Rice starch | 5 | 0.002 ± 0.050 |


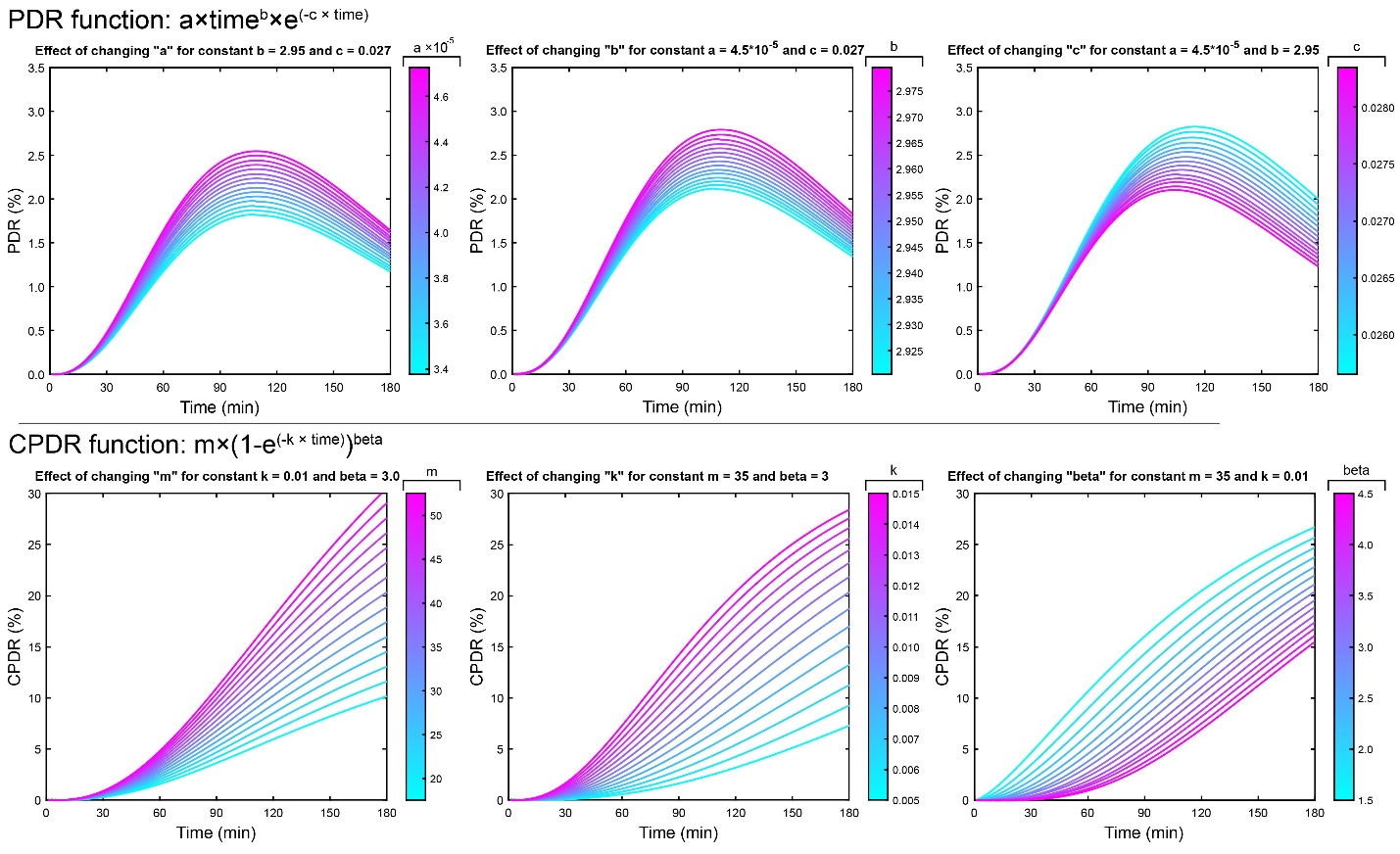


**Supplemental Figure 1**. Effect of changing one parameter at a time while holding the other two constant in the model for PDR (top row) and CPDR (bottom row).

# Results

## PDR and CPDR parameters from digestion of CP by healthy and stunted toddlers


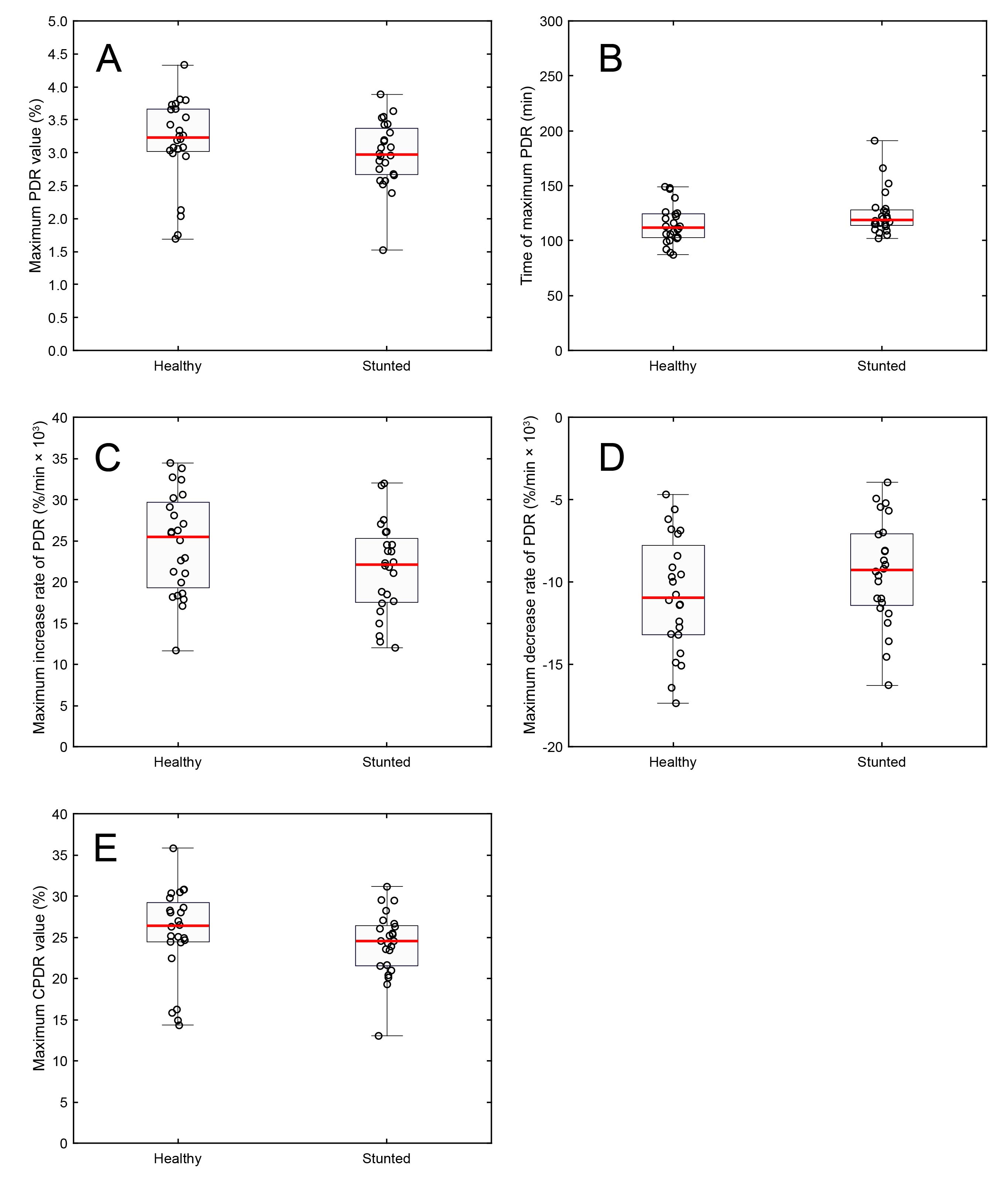


**Supplemental Figure 2**. Parameters from breath test analysis for the experiment comparing digestion of Common Sorghum Porridge (CP) in healthy and stunted Malian toddlers. Raw values are shown as black circles. The box extends from the 25^th^ to the 75^th^ percentile, with the whisker extending from the lowest to the highest observed value. The red horizontal line denotes the median observation.

## Parameters from curve fitting PDR and CPDR breath test curves

**Supplemental Table 3.** Parameters from curve fits to PDR (Equation 1) and CPDR (Equation 2) after consumption of CP (Common Sorghum Porridge) given as means ± standard deviation (standard error of the mean).

|  |  | **CP** | |
| --- | --- | --- | --- |
|  |  | **Healthy** | **Stunted** |
| **PDR**  **parameters** | **a×10^3^** | 0.41 ± 1.37 (0.28) | 0.19 ± 0.31 (0.06) |
|  | **b** | 3.01 ± 0.65 (0.13) | 2.94 ± 0.62 (0.13) |
|  | **c×10^2^** | 2.72 ± 0.83 (0.17) | 2.45 ± 0.71 (0.15) |
|  | **R^2^** | 0.99 ± 0.009 (0.002) | 0.993 ± 0.006 (0.001) |
| **CPDR parameters** | **m** | 42.88 ± 16.10 (3.29) | 45.34 ± 15.85 (3.23) |
|  | **k×10^2^** | 1.23 ± 0.35 (0.07) | 1.08 ± 0.29 (0.06) |
|  | **β** | 3.56 ± 0.67 (0.14) | 3.44 ± 0.63 (0.13) |
|  | **R^2^** | 1.00 ± 0.00 (0.000) | 1.00 ± 0.00 (0.000) |

**Supplemental Table 4.** Parameters from curve fits to PDR (Equation 1) and CPDR (Equation 2) after consumption of TP (energy dense thick porridge) and ATTP (energy dense, enzymatically thinned porridge) given as means ± standard deviation (standard error of the mean).

|  |  | **ATTP** | | **TP** | |
| --- | --- | --- | --- | --- | --- |
|  |  | **Healthy** | **Stunted** | **Healthy** | **Stunted** |
| **PDR**  **parameters** | **a×10^3^** | 0.16 ± 0.19 (0.04) | 0.41 ± 0.68 (0.14) | 0.12 ± 0.20 (0.04) | 0.3 ± 0.55 (0.12) |
|  | **b** | 2.80 ± 0.55 (0.11) | 2.55 ± 0.61 (0.12) | 2.92 ± 0.65 (0.13) | 2.61 ± 0.59 (0.13) |
|  | **c×10^2^** | 2.40 ± 0.57 (0.12) | 1.83 ± 0.67 (0.14) | 2.30 ± 0.98 (0.20) | 1.87 ±0.59 (0.13) |
|  | **R^2^** | 0.98 ± 0.054 (0.011) | 0.996 ± 0.005 (0.001) | 0.985 ± 0.05 (0.01) | 0.997 ± 0.002 (0.000) |
| **CPDR parameters** | **m** | 33.46 ± 16.21 (3.31) | 44.10 ± 21.51 (4.39) | 39.05 ± 17.99 (3.67) | 43.00 ± 14.99 (3.2) |
|  | **k×10^2^** | 1.12 ± 0.30 (0.06) | 0.83 ± 0.28 (0.06) | 1.01 ± 0.45 (0.09) | 0.85 ± 0.27 (0.06) |
|  | **β** | 3.44 ± 0.84 (0.17) | 3.16 ± 0.63 (0.13) | 3.49 ± 0.74 (0.15) | 3.24 ± 0.77 (0.16) |
|  | **R^2^** | 1.00 ± 0.001 (0.000) | 1.00 ± 0.00 (.000) | 1.00 ± 0.001 (0.000) | 1.00 ± 0.00 (0.000) |

**Supplemental Table 5.** Parameters from curve fits to PDR (Equation 1) and CPDR (Equation 2) after consumption of porridge with ^13^C-labelled algal starch (AS) and porridge with ^13^C-labelled algal limit dextrins (ALDx) given as means ± standard deviation (standard error of the mean).

|  |  | **AS** | | | **ALDx** | | |
| --- | --- | --- | --- | --- | --- | --- | --- |
|  |  | **Healthy** | **Stunted** | **US Children** | **Healthy** | **Stunted** | **US Children** |
| **PDR parameters** | **a×10^3^** | 0.31 ± 0.76 (0.16) | 0.66 ± 2.36 (0.48) | 1.54 ± 2.14 (0.88) | 0.24 ± 0.57 (0.12) | 0.78 ± 1.42 (0.3) | 0.28 ± 0.64 (0.26) |
|  | **b** | 2.79 ± 0.8 (0.16) | 2.74 ± 0.74 (0.15) | 2.21 ± 0.55 (0.23) | 3.14 ± 0.86 (0.17) | 2.40 ± 0.67 (0.14) | 2.72 ± 0.44 (0.18) |
|  | **c×10^2^** | 2.30 ± 1.05 (0.22) | 2.23 ± 0.66 (0.13) | 2.15 ± 0.49 (0.2) | 2.84 ± 0.91 (0.19) | 2.02 ± 0.85 (0.18) | 2.27 ± 0.42 (0.17) |
|  | **R^2^** | 0.99 ± 0.009 (0.002) | 0.98 ± 0.012 (0.002) | 0.99 ± 0.004 (0.002) | 0.99 ± 0.014 (0.003) | 0.98 ± 0.036 (0.007) | 0.99 ± 0.003 (0.001) |
| **CPDR parameters** | **m** | 19.53 ± 15.19 (3.1) | 14.05 ± 5.55 (1.13) | 14.79 ± 4.26 (1.74) | 18.19 ± 7.98 (1.63) | 25.39 ± 19.27 (4.02) | 14.69 ± 3.93 (1.61) |
|  | **k×10^2^** | 1.03 ± 0.47 (0.1) | 1.00 ± 0.27 (0.05) | 1.10 ± 0.24 (0.1) | 1.26 ± 0.39 (0.08) | 0.94 ± 0.39 (0.08) | 1.07 ± 0.27 (0.11) |
|  | **β** | 3.33 ± 0.79 (0.16) | 3.29 ± 0.99 (0.2) | 2.81 ± 0.54 (0.22) | 3.71 ± 1.06 (0.22) | 2.94 ± 0.64 (0.13) | 3.29 ± 0.48 (0.2) |
|  | **R^2^** | 1.00 ± 0.00 (0.000) | 1.00 ± 0.00 (0.000) | 1.00 ± 0.00 (0.000) | 1.00 ± 0.00 (0.000) | 1.00 ± 0.00 (0.000) | 1.00 ± 0.00 (0.000) |

**Supplemental Table 6.** Parameters from curve fits to PDR (Equation 1) and CPDR (Equation 2) after consumption of OCT (porridge with ^13^C-labelled octanoic acid for assessment of gastric emptying) given as means ± standard deviation (standard error of the mean).

|  |  | **OCT** | |
| --- | --- | --- | --- |
|  |  | **Healthy** | **Stunted** |
| **PDR**  **parameters** | **a×10^3^** | 93.32 ± 163.92 (33.46) | 78.45 ± 110.73 (22.6) |
|  | **b** | 1.65 ± 1.00 (0.2) | 1.37 ± 0.55 (0.11) |
|  | **c×10^2^** | 2.89 ± 1.75 (0.36) | 2.39 ± 0.91 (0.19) |
|  | **R^2^** | 0.925 ±0.067 (0.014) | 0.923 ± 0.06 (0.012) |
| **CPDR parameters** | **m** | 19.36 ± 11.01 (2.25) | 23.09 ± 10.39 (2.12) |
|  | **k×10^2^** | 1.84 ± 1.44 (0.29) | 1.46 ± 0.53 (0.11) |
|  | **β** | 2.67 ± 2.44 (0.5) | 1.98 ± 0.52 (0.11) |
|  | **R^2^** | 0.998 ± 0.003 (0.001) | 0.998 ± 0.004 (0.001) |

## Significance levels of factors

**Supplemental Table 7**. Significance level of factors from analysis of breath test parameters from the experiment with common sorghum porridge (CP). One-factor ANOVA models analyzed only the effect of participant stunting status (stunted, healthy) whereas two-factor ANOVA models analyzed the effect of participant status as well as participant sex (male, female). Cells with p value > 0.05 (not significant) are highlighted in red.

|  | **One-factor ANOVA** |  | **Two-factor ANOVA** | | |
| --- | --- | --- | --- | --- | --- |
|  | **status** |  | **status** | **sex** | **status*sex** |
| a | 0.9515 |  | 0.9813 | 0.4447 | 0.5862 |
| b | 0.7211 |  | 0.8611 | 0.0195 | 0.2412 |
| c | 0.2353 |  | 0.3004 | 0.0301 | 0.2537 |
| Maximum PDR value | 0.0995 |  | 0.0735 | 0.0014 | 0.27 |
| Time of maximum PDR | 0.0626 |  | 0.0463 | 0.2531 | 0.4752 |
| Maximum increase rate of PDR | 0.0775 |  | 0.0834 | 0.0014 | 0.2018 |
| Maximum decrease rate of PDR | 0.1593 |  | 0.1829 | 0.0014 | 0.1852 |
| m | 0.7608 |  | 0.5716 | 0.8548 | 0.7013 |
| k | 0.1111 |  | 0.1523 | 0.056 | 0.2326 |
| β | 0.5435 |  | 0.6726 | 0.0242 | 0.2134 |
| Maximum CPDR value | 0.1393 |  | 0.117 | 0.0057 | 0.2561 |

**Supplemental Table 8**. Significance level of factors from analysis of breath test parameters from the experiment with modified sorghum porridge (porridge with additional starch, TP) and modified thinned porridge (porridge with additional starch that was also processed by enzymatic thinning, ATTP). Two-factor ANOVA models analyzed the effect of participant stunting status (stunted, healthy) and porridge type (TP vs ATTP) whereas three-factor ANOVA models analyzed the effect of participant stunting status and porridge type as well as participant sex (male, female). Cells with p value > 0.05 (not significant) are highlighted in red.

|  | **Two-factor ANOVA** | | | **Three-factor ANOVA** | | | | | | |
| --- | --- | --- | --- | --- | --- | --- | --- | --- | --- | --- |
|  | **porridge** | **status** | **porridge**  ***status** | **porridge** | **status** | **porridge**  ***status** | **sex** | **porridge**  ***sex** | **status**  ***sex** | **porridge**  ***status**  ***sex** |
| a | 0.2337 | 0.1229 | 0.9934 | 0.2766 | 0.0219 | 0.6781 | 0.0473 | 0.3595 | 0.0447 | 0.3131 |
| b | 0.3994 | 0.0378 | 0.8381 | 0.2835 | 0.0291 | 0.6169 | 0.0005 | 0.7702 | 0.2358 | 0.5386 |
| c | 0.8220 | 0.0012 | 0.6334 | 0.9211 | 0.0006 | 0.6983 | 0.0001 | 0.4527 | 0.8631 | 0.4184 |
| Maximum PDR value | 0.7010 | 0.7899 | 0.5052 | 0.6070 | 0.7287 | 0.4228 | 0.0102 | 0.3515 | 0.9314 | 0.4468 |
| Time of maximum PDR | 0.3044 | 0.0017 | 0.1506 | 0.1199 | 0.0022 | 0.0861 | 0.0014 | 0.4558 | 0.5698 | 0.3831 |
| Maximum increase rate of PDR | 0.8085 | 0.0714 | 0.1926 | 0.8483 | 0.0457 | 0.1807 | 0.0000 | 0.8675 | 0.3342 | 0.7259 |
| Maximum decrease rate of PDR | 0.8732 | 0.0402 | 0.2734 | 0.9537 | 0.0200 | 0.3318 | 0.0000 | 0.8698 | 0.4918 | 0.8146 |
| m | 0.4698 | 0.0887 | 0.5876 | 0.4599 | 0.0716 | 0.5000 | 0.4511 | 0.3646 | 0.8591 | 0.6051 |
| k | 0.5382 | 0.0014 | 0.3641 | 0.6181 | 0.0012 | 0.3883 | 0.0008 | 0.4605 | 0.8760 | 0.5911 |
| β | 0.3591 | 0.0985 | 0.7451 | 0.4026 | 0.0561 | 0.4829 | 0.0039 | 0.8895 | 0.1009 | 0.8742 |
| Maximum CPDR value | 0.8513 | 0.7963 | 0.2632 | 0.9538 | 0.8115 | 0.2508 | 0.0131 | 0.5385 | 0.8638 | 0.5643 |

**Supplemental Table 9**. Significance level of factors from analysis of breath test parameters from the experiment with common sorghum porridge with labeled algal starch (AS) and common porridge with labeled algal limit dextrins (ALDx). Two-factor ANOVA models analyzed the effect of participant stunting status (stunted, healthy) and porridge type (AS vs ALDx) whereas three-factor ANOVA models analyzed the effect of participant stunting status and porridge type as well as participant sex (male, female). Cells with p value > 0.05 (not significant) are highlighted in red.

|  | **Two-factor ANOVA** | | | **Three-factor ANOVA** | | | | | | |
| --- | --- | --- | --- | --- | --- | --- | --- | --- | --- | --- |
|  | **porridge** | **status** | **porridge**  ***status** | **porridge** | **status** | **porridge**  ***status** | **sex** | **porridge**  ***sex** | **status**  ***sex** | **porridge**  ***status**  ***sex** |
| a | 0.8481 | 0.0392 | 0.0053 | 0.3684 | 0.3499 | 0.2628 | 0.4097 | 0.7107 | 0.9626 | 0.4150 |
| b | 0.3259 | 0.0168 | 0.0472 | 0.3562 | 0.0145 | 0.0667 | 0.4075 | 0.8402 | 0.5680 | 0.5433 |
| c | 0.452 | 0.0398 | 0.103 | 0.5032 | 0.0333 | 0.1310 | 0.8842 | 0.7968 | 0.1794 | 0.7454 |
| Maximum PDR value | 0.0001 | 0.0356 | 0.0284 | 0.0185 | 0.0980 | 0.1464 | 0.2126 | 0.7739 | 0.5032 | 0.9231 |
| Time of maximum PDR | 0.1905 | 0.72 | 0.0623 | 0.2191 | 0.3335 | 0.0339 | 0.9590 | 0.6104 | 0.1002 | 0.9199 |
| Maximum increase rate of PDR | 0.0471 | 0.1132 | 0.0206 | 0.0435 | 0.0756 | 0.0193 | 0.5501 | 0.7716 | 0.1346 | 0.8467 |
| Maximum decrease rate of PDR | 0.0028 | 0.0512 | 0.0745 | 0.0042 | 0.0433 | 0.0657 | 0.0417 | 0.7643 | 0.0769 | 0.9689 |
| m | 0.0204 | 0.3559 | 0.098 | 0.0070 | 0.1823 | 0.0239 | 0.8525 | 0.4083 | 0.3810 | 0.7904 |
| k | 0.4013 | 0.089 | 0.0684 | 0.3064 | 0.0870 | 0.0828 | 0.3667 | 0.7154 | 0.0290 | 0.9207 |
| β | 0.5431 | 0.0324 | 0.1116 | 0.8261 | 0.0267 | 0.0963 | 0.1974 | 0.8835 | 0.5989 | 0.5055 |
| Maximum CPDR value | 0.0252 | 0.4589 | 0.0578 | 0.0265 | 0.3735 | 0.0609 | 0.2127 | 0.6430 | 0.6017 | 0.8120 |

**Supplemental Table 10.** Significance level of factors from analysis of breath test parameters from the gastric emptying assessment (Section 3.10) using modified sorghum porridge with labeled octanoic acid (OCT). One-factor ANOVA models analyzed only the effect of participant stunting status (stunted, healthy) whereas two-factor ANOVA models analyzed the effect of participant status as well as participant sex (male, female). Cells with p value > 0.05 (not significant) are highlighted in red.

|  | **One-factor ANOVA** |  | **Two-factor ANOVA** | | |
| --- | --- | --- | --- | --- | --- |
|  | status |  | status | sex | status*sex |
| $T_{1/2}$ | 0.9000 |  | 0.7114 | 0.8563 | 0.6833 |
| $T_{lag}$ | 0.7375 |  | 0.5687 | 0.4045 | 0.6526 |
| $GEC$ | 0.6002 |  | 0.4260 | 0.1275 | 0.8177 |

## CP





**Supplemental Figure 3**. All breath test curves (PDR and CPDR) for digestion of CP by healthy Malian toddlers.





**Supplemental Figure 4**. All breath test curves (PDR and CPDR) for digestion of CP by stunted Malian toddlers.

## TP





**Supplemental Figure 5.** All breath test curves (PDR and CPDR) for digestion of TP by healthy Malian toddlers.





**Supplemental Figure 6**. All breath test curves (PDR and CPDR) for digestion of TP by stunted Malian toddlers.

## ATTP





**Supplemental Figure 7**. All breath test curves (PDR and CPDR) for digestion of ATTP by healthy Malian toddlers.





**Supplemental Figure 8**. All breath test curves (PDR and CPDR) for digestion of ATTP by stunted Malian toddlers. Two outliers were identified and are shown in the red boxes.

## AS





**Supplemental Figure 9**. All breath test curves (PDR and CPDR) for digestion of AS by healthy Malian toddlers.





**Supplemental Figure 10**. All breath test curves (PDR and CPDR) for digestion of AS by stunted Malian toddlers.


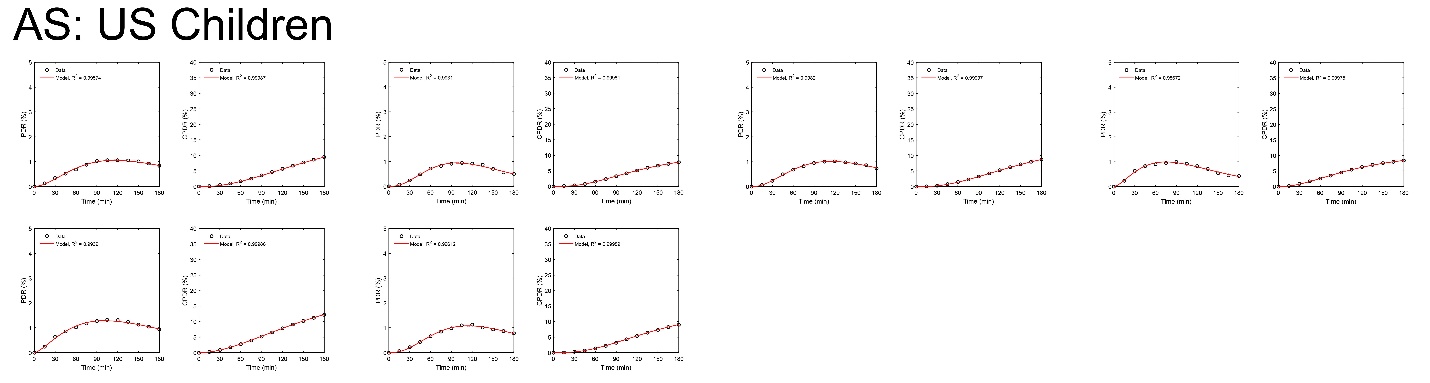


**Supplemental Figure 11**. All breath test curves (PDR and CPDR) for digestion of AS by US children.

## ALDx





**Supplemental Figure 12**. All breath test curves (PDR and CPDR) for digestion of ALDx by healthy Malian toddlers.





**Supplemental Figure 13**. All breath test curves (PDR and CPDR) for digestion of ALDx by stunted Malian toddlers. One outlier was identified and is shown in the red box.


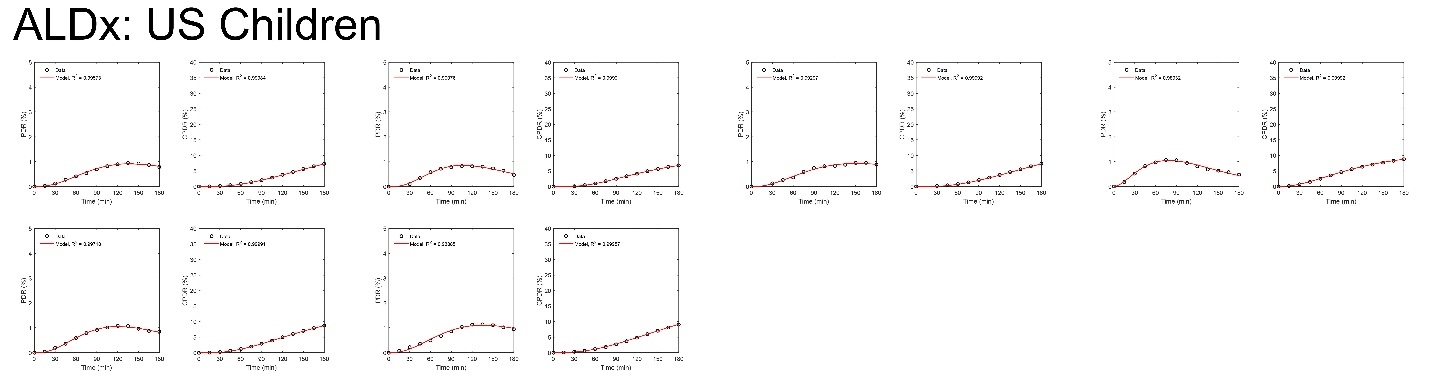


**Supplemental Figure 14**. All breath test curves (PDR and CPDR) for digestion of ALDx by US children.

## OCT





**Supplemental Figure 15**. All breath test curves (PDR and CPDR) for digestion of OCT (gastric emptying assessment) by healthy Malian toddlers. One outlier was identified and is shown in the red box.


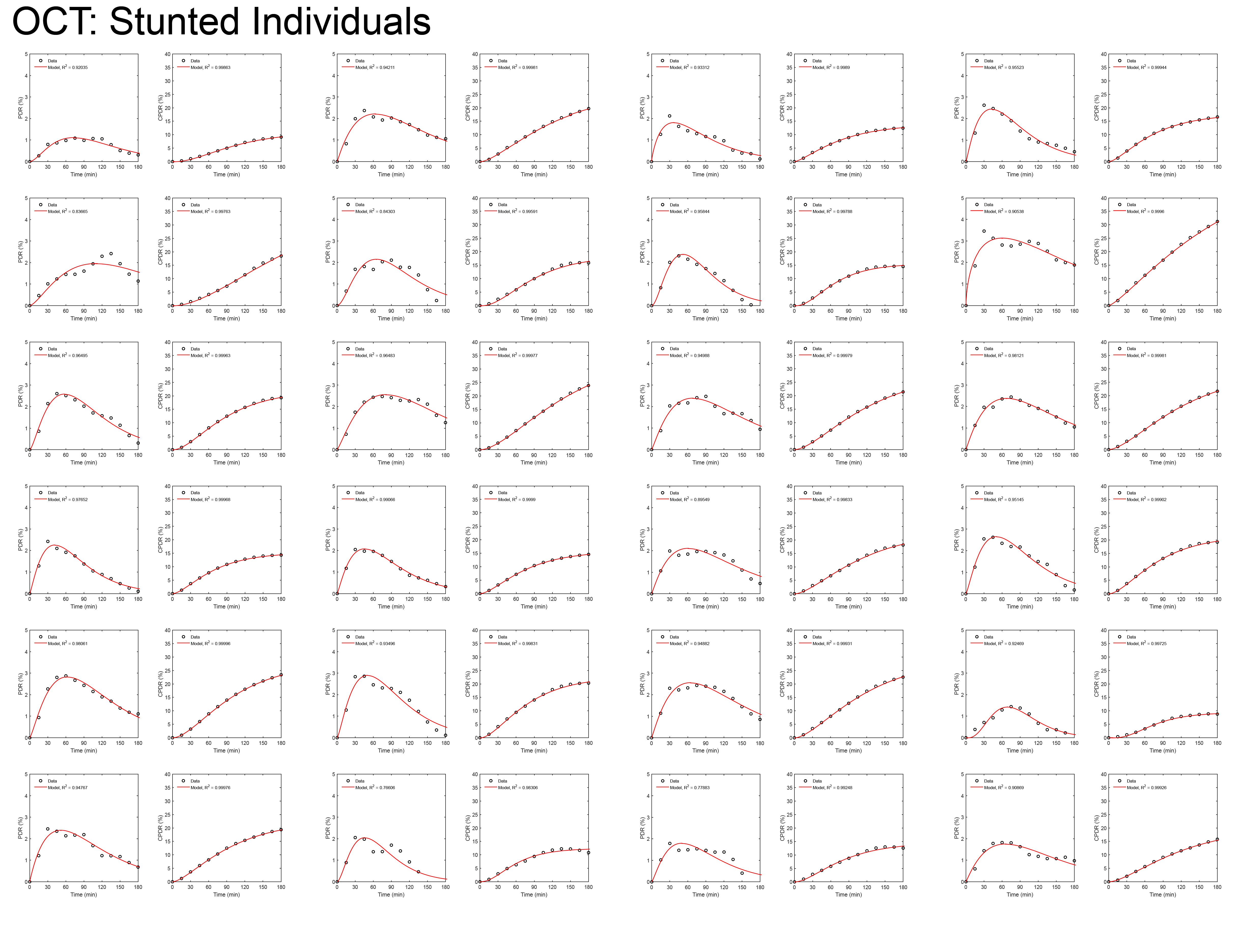


**Supplemental Figure 16**. All breath test curves (PDR and CPDR) for digestion of OCT (gastric emptying assessment) by stunted Malian toddlers.

# References

Amarri, S., Weaver, L.T., 1995. 13C-breath tests to measure fat and carbohydrate digestion in clinical practice. Clin. Nutr. 14, 149–154. https://doi.org/10.1016/S0261-5614(95)80012-3

Haycock, G.B., Schwartz, G.J., Wisotsky, D.H., 1978. Geometric method for measuring body surface area: A height-weight formula validated in infants, children, and adults. J. Pediatr. 93, 62–66. https://doi.org/10.1016/S0022-3476(78)80601-5

Shreeve, W.W., Cerasi, E., Luft, R., 1970. Metabolism of [2-14C] pyruvate in normal, acromegalic and HGH-treated human subjects. Acta Endocrinol. (Copenh). 65, 155–169. https://doi.org/10.1530/acta.0.0650155
